# Transient GI/MSP/1/N Queue

**DOI:** 10.3390/e26090807

**Published:** 2024-09-22

**Authors:** Andrzej Chydzinski

**Affiliations:** Department of Computer Networks and Systems, Silesian University of Technology, Akademicka 16, 44-100 Gliwice, Poland; andrzej.chydzinski@polsl.pl

**Keywords:** single-server queue, correlated service times, Markovian service process, GI/MSP/1/N queue, transient analysis, queue length distribution, mean queue length

## Abstract

A non-zero correlation between service times can be encountered in many real queueing systems. An attractive model for correlated service times is the Markovian service process, because it offers powerful fitting capabilities combined with analytical tractability. In this paper, a transient study of the queue length in a model with MSP services and a general distribution of interarrival times is performed. In particular, two theorems are proven: one on the queue length distribution at a particular time *t*, where *t* can be arbitrarily small or large, and another on the mean queue length at *t*. In addition to the theorems, multiple numerical examples are provided. They illustrate the development over time of the mean queue length and the standard deviation, along with the complete distribution, depending on the service correlation strength, initial system conditions, and the interarrival time variance.

## 1. Introduction

In everyday applications of queueing systems, service times are often correlated. One possible cause of this is the arrival of many customers with similar characteristics due to some external event. For instance, if there is a fire in a town, many patients with similar needs may enter the hospital’s emergency room one after another, requiring similar treatment. If there is a sale in a supermarket, many customers may want to purchase the same item. If a company’s specific service breaks down (e.g., no Internet signal from an ISP), its call center may expect many calls of the same type, and so on.

Correlated service times can be modeled mathematically in various ways. One of the most attractive methods is to use the Markovian service process (MSP). This is because any service time distribution can be closely mimicked by a properly parameterized MSP, which simultaneously can have a very well-fitted correlation function. Moreover, these powerful modeling capabilities do not make the MSP analytically intractable.

For these and other reasons, several papers on queueing systems with MSP service have been published, addressing specific performance metrics of the system (e.g., queue length, waiting time, blocking probability, etc.) under different assumptions about the organization of the system (e.g., finite vs. infinite waiting room) and the arrival process (Poisson, general renewal, batch arrivals, etc.). However, all of this work has been dedicated to stationary analysis only, i.e., when the queue has reached equilibrium and the influence of the initial conditions has vanished.

In this paper, we perform a transient analysis of a queue with MSP service. Specifically, we obtain the queue length distribution and the mean queue length at a specific time *t*, where *t* can be arbitrarily small or large. This allows for a much deeper insight into the system’s performance than stationary distribution alone provides. For instance, we can examine the impact of a specific initial occupancy of the waiting room on the queue development shortly after the system starts, and we can determine how long it takes to reach stationary performance after the system starts, among other things. Moreover, the transient results obtained here incorporate the stationary results. Specifically, we can use t=∞ in the formulas to obtain the stationary distribution. All of these points are illustrated in numerical examples.

To make the considered model broadly applicable, the arrival process here has the form of the general renewal process, i.e., the interarrival time has arbitrary distribution.

Summarizing, the original contribution of the paper consist of:

- A theorem on the queue length distribution at time *t*;
- A theorem on the mean queue length at time *t*;
- Numerical calculations carried out for three distinct parameterizations of the MSP and two arrival processes. They illustrate the development over time of the mean queue length, along with its standard deviation and complete distribution, depending on the service correlation strength, initial system conditions, and the interarrival distribution.

These theorems are proven for a system with MSP service, a general interarrival distribution, and a finite waiting room of capacity *N*, i.e., for the GI/MSP/1/N system in Kendall’s notation.

A finite waiting room is assumed for several reasons. In the majority of real systems, the waiting room capacity has a practical limit. This is true with no exceptions for all electronic and computer devices, though not exclusively. The performance of an infinite waiting room can be approximated by using a large waiting room capacity, but not vice versa. Lastly, a finite waiting room induces customer loss, i.e., customers who arrive when the waiting room is saturated and leave the system unserved. This phenomenon significantly impacts system performance but is absent in models with an infinite waiting room.

The remaining content of the paper is laid out as follows. In Section 2, a literature review on queueing models with MSP service is given. In Section 3, the system model is formalized, including the definition of the Markovian service process. Then, in Section 4, the two main theorems are proven: one on the queue length distribution at *t*, and another on the mean queue length at *t*. Section 5 includes the derivation of some intermediate characteristics that appear in the two mentioned theorems. The formulas for these intermediate characteristics are needed to make the theorems entirely applicable in numeric calculations. In Section 6, numeric outcomes are shown and examined. For three parameterizations of the MSP and two arrival processes, we observe progress over time of the queue distribution, its mean value, and its standard deviation. Among other aspects, we discuss the time needed to reach the stationary regime depending on the service correlation strength, the interarrival time variance, along with the effect of initial system conditions on the development of the queue length. In Section 7, concluding remarks are presented.

## 2. Related Work

To the author’s awareness, the findings shown here are new. Specifically, the literature on queueing systems with MSP service has focused on stationary analysis so far. All the results mentioned in the next paragraphs were obtained in the stationary regime only.

In [1], the queue length distribution in a system with finite waiting rooms was obtained. In [2], the tail of the queue in a system with an infinite waiting room was studied. In [3], several other characteristics were investigated, including the queue length at arrival times, the sojourn time distribution, and the waiting time distribution, for both versions of the system—with finite and infinite waiting rooms. In [4], an expanded version of the service policy, allowing batch services, was studied in a system with a finite waiting room. For such a system, the queue length and sojourn time distributions are derived. Ref. [5] deals with the multi-server queue with MSP service and a finite waiting room. For this system, the queue length distribution, busy period distribution (when all service stations are occupied), and partly-idle period distribution (when not all service stations are occupied) are obtained. In [6], the waiting time and queue length distributions were studied under a slightly different formulation of MSP, where the service process starts with the same predefined initial phase distribution after every idle period. In [7], the asymptotic behavior of the loss probability is shown as the waiting room capacity grows to infinity. Ref. [8] is devoted to an expanded version of the arrival process, incorporating batch arrivals. Moreover, two batch acceptance policies are studied: partial batch rejection and total batch rejection. The queue length distribution, along with the waiting time distribution, loss probability, and the probability of blocking the first and last customer of a batch are derived there.

In [9], the system with batch services was studied again as in [4], but assuming an infinite waiting room. The queue length distribution, queue length distribution at arrival time, and mean sojourn time are calculated. In [10], a different approach, based on the roots of the characteristic equation, was used to acquire the queue length distribution for a queue with an infinite waiting room. In [11], the sojourn time distribution and the correlation coefficient of interdeparture intervals were obtained under the infinite waiting room assumption. In works [12,13], a system with batch arrivals and an infinite waiting room was investigated. Specifically, [12] focused on the queue length, along with the busy and idle periods, while [13] focused on the waiting time distribution. Ref. [14] also deals with a batch-arrival system, but with a finite waiting room. For such a system, the following characteristics are computed: blocking probability, mean waiting time of the first, last, and arbitrary customer in a batch, and the probability of *k* or more consecutive losses in a busy period. In [15], a system with a finite waiting room and batch service is examined again. The study is directed at optimizing a profit function of the system, which incorporates several key characteristics, including the mean queue length, mean waiting time, and blocking probability. Finally, in [16], queue length distributions for two different models of the batch MSP are compared. In the first model, which is commonly used and also adopted here, the service phase does not change during the idle period. In the second model, the service phase evolves during the idle period.

The literature review presented in the previous paragraphs incorporates only models where the interarrival distribution is of a general type. Other works with MSP service can be found, where the interarrival time is of some specific type, e.g., Poisson, batch Poisson, MAP, MMPP, and others. Moreover, the presented review is devoted to continuous queueing models, as is the case considered herein. There are also some works on discrete-time models of MSP (i.e., D-MSP, D-BMSP).

## 3. Queue Model

We focus on the GI/MSP/1/N system in Kendall’s notation. Specifically, arrivals constitute a general renewal process, with mutually independent interarrival times distributed in accordance with the distribution function G(t), of a finite mean. The service times are of the MSP type, which will be detailed below. There is one service station serving customers from the queue. The waiting room has a finite capacity *N*. If at the moment of arrival the waiting room is saturated, the new customer leaves the queueing system unserved. We operate under the convention that the number *N* includes the service position.

The specific queueing discipline does not impact the theorems proven here, so any discipline can be assumed, e.g., FIFO or LIFO, as long as it does not require interrupting a service already in progress.

To define the Markovian service process, (see, e.g., [3]), we need square matrices $L_{0}$ and $L_{1}$, each of M×M size, where $L_{0}$ has negative diagonal entries and non-negative off-diagonal entries, $L_{1}$ has only non-negative entries, whereas $L_{0}+L_{1}$ has zero sum of each row.

When the system is not vacant, the development of the MSP is governed by a CTMC (continuous-time Markov chain) with the rate matrix $L_{0}+L_{1}$ and states {1,…,M}, called “service phases”. In detail, if the service phase at some *t* is *i*, then shortly after *t*, i.e., at t+Δ, the phase may change to *j* with the probability ${\left(L_{0}\right)}_{ij}\Delta +o\left(\Delta \right)$, and the current service of a customer is carried on, or the phase may change to *j* with the probability ${\left(L_{1}\right)}_{ij}\Delta +o\left(\Delta \right)$, and the current service of a customer is completed, so the customer departs the system.

When the system becomes empty after a completed service, the service phase stops evolving and remains the same during the whole idle period. After that, upon a new arrival, it continues evolving according to the described CTMC.

The key parameter of the MSP, i.e., the service rate μ, can be computed as:

(1) $$\mu =\pi L_{1}1,$$

where π is the sole solution of $\pi (L_{0}+L_{1})=0$, π1=1. Here, **0** is the vector of zeros, while 1 is the column vector of ones.

By X(t), we denote the queue length (system occupancy) at *t*, comprising the service position, if taken. By S(t), we denote the service phase at *t*. Pay attention that S(t) is not a CTMC, because during the idle periods S(t) remains constant and does not follow the CTMC development.

We assume also that the arrival process is not shifted at t=0, i.e., the first arrival time is distributed in accordance with G(t).

## 4. Analysis

### 4.1. Queue 0 Distribution

Our chief target is to calculate the probability that the queue has length *n* at time *t*, given that at the time origin the queue length was *m* and the service phase was *j*.

Namely, we want to derive:

(2) $$P_{j}\left(t,m,n\right)=P\left\{X\left(t\right)=n|X\left(0\right)=m,S\left(0\right)=j\right\},\;\;\;\;n,m=0,\ldots ,N;j=1,\ldots ,M.$$

Firstly, we need to define two important functions: $f_{jk}\left(v,l\right)$ and $r_{jk}\left(v,l\right)$. Specifically, $f_{jk}\left(v,l\right)$ is the probability that *l* services will be completed in a time interval of duration *v*, and the service phase will be *k* at the close of this interval, given that at the start of this interval, the queue length was no less than *l* and the service phase was *j*. Then, $r_{jk}\left(v,l\right)$ is the probability that *l* services will be completed in a time interval of duration *v*, and the service phase will be *k* at the *l*-th service completion epoch, given that at the start of this interval, the queue length was no less than *l* and the service phase was *j*.

For any m=1,…,N−1 and any *j*, we can obtain the following integral equation for $P_{j}\left(t,m,n\right)$:

(3) $$\begin{array}{cc} P_{j}\left(t,m,n\right)=\; & \sum_{l=0}^{m-1}\sum_{k=1}^{M}\int_{0}^{t}f_{jk}\left(u,l\right)P_{k}\left(t-u,m-l+1,n\right)dG\left(u\right)\\ \; & +\sum_{k=1}^{M}\int_{0}^{t}r_{jk}\left(u,m\right)P_{k}\left(t-u,1,n\right)dG\left(u\right)\\ \; & +\left[1-G\left(t\right)\right]\left[\delta_{1\leq n\leq m}\sum_{k=1}^{M}f_{jk}\left(t,m-n\right)+\delta_{n=0}\sum_{l=m}^{\infty }\sum_{k=1}^{M}f_{jk}\left(t,l\right)\right], \end{array}$$

where $\delta_{A}$ denotes the indicator function such that $\delta_{x}=1$, if *x* holds, while $\delta_{x}=0$, if *x* does not hold.

Equation (3) is crafted conditioning on the first arrival time, *u*. Specifically, the first component covers the case where u<t and less than *m* services were completed by *u*, so the MSP does not stop evolving at *u*. The second component of (3) covers the case where u<t and *m* services were completed by *u*, so the MSP stops evolving before *u* at phase *k*, and this phase is preserved until *u*. The third component of (3) covers the case where u≥t.

Assuming m=0 and any *j*, we get the following integral equation for $P_{j}\left(t,0,n\right)$:

(4) $$\begin{array}{c} P_{j}\left(t,0,n\right)=\int_{0}^{t}P_{j}\left(t-u,1,n\right)dG\left(u\right)+\delta_{n=0}\left[1-G\left(t\right)\right]. \end{array}$$

Equation (4) is obtained conditioning again on the first arrival time, *u*. The first part covers the case u<t, while the second covers the case u≥t.

For m=N and any *j*, we obtain the equation for $P_{j}\left(t,N,n\right)$ as follows:

(5) $$\begin{array}{cc} P_{j}\left(t,N,n\right)=\; & \sum_{k=1}^{M}\int_{0}^{t}f_{jk}\left(u,0\right)P_{k}\left(t-u,N,n\right)dG\left(u\right)\\ \; & +\sum_{l=1}^{N-1}\sum_{k=1}^{M}\int_{0}^{t}f_{jk}\left(u,l\right)P_{k}\left(t-u,N-l+1,n\right)dG\left(u\right)\\ \; & +\sum_{k=1}^{M}\int_{0}^{t}r_{jk}\left(u,N\right)P_{k}\left(t-u,1,n\right)dG\left(u\right)\\ \; & +\left[1-G\left(t\right)\right]\left[\delta_{1\leq n\leq N}\sum_{k=1}^{M}f_{jk}\left(t,N-n\right)+\delta_{n=0}\sum_{l=N}^{\infty }\sum_{k=1}^{M}f_{jk}\left(t,l\right)\right]. \end{array}$$

As we can notice, (5) is similar to (3), but not identical. The difference is that in the first part of (5) we have to single out the case l=0, under which the new queue at *u* is *N*, not N+1, which is impossible in the system of interest. The rest of the design of (5) is the same as in (3).

Now, we will solve the system (3), (4) and (5) using the Laplace transform method and some recursive sequences of matrices.

Denote:

(6) $$p_{j}\left(s,m,n\right)=\int_{0}^{\infty }e^{-st}P_{j}\left(t,m,n\right)dt,$$

(7) $$g\left(s\right)=\int_{0}^{\infty }e^{-st}dG\left(t\right),$$

(8) $$a_{jk}\left(s,l\right)=\int_{0}^{\infty }e^{-st}f_{jk}\left(t,l\right)dG\left(t\right),$$

(9) $$b_{jk}\left(s,l\right)=\int_{0}^{\infty }e^{-st}r_{jk}\left(t,l\right)dG\left(t\right),$$

(10) $$c_{jk}\left(s,l\right)=\int_{0}^{\infty }e^{-st}f_{jk}\left(t,l\right)\left[1-G(t)\right]dt.$$

For m=1,…,N−1, Equation (3) yields:

(11) $$\begin{array}{cc} p_{j}\left(s,m,n\right)=\; & \sum_{l=0}^{m-1}\sum_{k=1}^{M}a_{jk}\left(s,l\right)p_{k}\left(s,m-l+1,n\right)+\sum_{k=1}^{M}b_{jk}\left(s,m\right)p_{k}\left(s,1,n\right)\\ \; & +\delta_{1\leq n\leq m}\sum_{k=1}^{M}c_{jk}\left(s,m-n\right)+\delta_{n=0}\left[1-g\left(s\right)\right]s^{-1}-\delta_{n=0}\sum_{l=0}^{m-1}\sum_{k=1}^{M}c_{jk}\left(s,l\right). \end{array}$$

The last two components of (11) are obtained from the last component of (3) using the following relations:

(12) $$\sum_{l=m}^{\infty }\sum_{k=1}^{M}f_{jk}\left(t,l\right)=\sum_{l=0}^{\infty }\sum_{k=1}^{M}f_{jk}\left(t,l\right)-\sum_{l=0}^{m-1}\sum_{k=1}^{M}f_{jk}\left(t,l\right)=1-\sum_{l=0}^{m-1}\sum_{k=1}^{M}f_{jk}\left(t,l\right).$$

From (4), we obtain

(13) $$\begin{array}{c} p_{j}\left(s,0,n\right)=g\left(s\right)p_{j}\left(s,1,n\right)+\delta_{n=0}\left[1-g\left(s\right)\right]s^{-1}, \end{array}$$

whereas (5) yields:

(14) $$\begin{array}{cc} p_{j}\left(s,N,n\right)=\; & \sum_{k=1}^{M}a_{jk}\left(s,0\right)p_{k}\left(s,N,n\right)+\sum_{l=1}^{N-1}\sum_{k=1}^{M}a_{jk}\left(s,l\right)p_{k}\left(s,N-l+1,n\right)\\ \; & +\sum_{k=1}^{M}b_{jk}\left(s,N\right)p_{k}\left(s,1,n\right)+\delta_{1\leq n\leq N}\sum_{k=1}^{M}c_{jk}\left(s,N-n\right)+\delta_{n=0}\left[1-g\left(s\right)\right]s^{-1}\\ \; & -\delta_{n=0}\sum_{l=0}^{N-1}\sum_{k=1}^{M}c_{jk}\left(s,l\right). \end{array}$$

Using matrices and vectors:

(15) $$A\left(s,l\right)={\left[a_{jk}\left(s,l\right)\right]}_{j=1,\ldots ,M;k=1,\ldots ,M},$$

(16) $$B\left(s,l\right)={\left[b_{jk}\left(s,l\right)\right]}_{j=1,\ldots ,M;k=1,\ldots ,M},$$

(17) $$C\left(s,l\right)={\left[c_{jk}\left(s,l\right)\right]}_{j=1,\ldots ,M;k=1,\ldots ,M},$$

(18) $$p\left(s,m,n\right)={\left[p_{1}\left(s,m,n\right),\ldots ,p_{M}\left(s,m,n\right)\right]}^{T},$$

from (11) we get for m=1,…,N−1:

(19) $$\begin{array}{c} p\left(s,m,n\right)=\sum_{l=0}^{m-1}A\left(s,l\right)p\left(s,m-l+1,n\right)+B\left(s,m\right)p\left(s,1,n\right)+y\left(s,m,n\right), \end{array}$$

where

(20) $$y\left(s,m,n\right)=\delta_{1\leq n\leq m}C\left(s,m-n\right)1+\delta_{n=0}\left[1-g\left(s\right)\right]s^{-1}1-\delta_{n=0}\sum_{l=0}^{m-1}C\left(s,l\right)1.$$

Similarly, (13) yields:

(21) $$\begin{array}{c} p(s,0,n)=g(s)p(s,1,n)+w(s,n), \end{array}$$

with

(22) $$w\left(s,n\right)=\delta_{n=0}\left[1-g\left(s\right)\right]s^{-1}1,$$

whereas (14) gives:

(23) $$\begin{array}{c} p\left(s,N,n\right)=A\left(s,0\right)p\left(s,N,n\right)+\;\sum_{l=1}^{N-1}\;A\left(s,l\right)p\left(s,N-l+1,n\right)+B\left(s,N\right)p\left(s,1,n\right)+y\left(s,N,n\right). \end{array}$$

Now, we can derive an explicite solution of the system (19), (21) and (23), by means of Lemma 3.2.1 of [17].

Specifically, (19) can be rewritten as:

(24) $$\sum_{l=0}^{m}A\left(s,l\right)p\left(s,m-l+1,n\right)-p\left(s,m,n\right)=\psi \left(s,m,n\right),\;\;\;m=1,\ldots ,N-1,$$

with

(25) ψ(s,m,n)=[A(s,m)−B(s,m)]p(s,1,n)−y(s,m,n).

According to Lemma 3.2.1 of [17], every solution of (24) can be expressed in the form:

(26) $$p\left(s,m,n\right)=R\left(s,m\right)c\left(s,n\right)+\sum_{k=1}^{m}R\left(s,m-k\right)\psi \left(s,k,n\right),$$

where

(27) $$R\left(s,0\right)=0,\;\;\;\;R\left(s,1\right)=A^{-1}\left(s,0\right),$$

(28) $$R\left(s,k+1\right)=R\left(s,1\right)\left[R\left(s,k\right)-\sum_{i=0}^{k}A\left(s,i+1\right)R\left(s,k-i\right)\right],$$

where 0 is the square zero matrix and c(s,n) is a vector independent of *m*.

Now, Formula (26) with m=1 gives:

(29) c(s,n)=A(s,0)p(s,1,n).

Hence, denoting:

(30) $$X\left(s,m\right)=R\left(s,m\right)A\left(s,0\right)+\sum_{k=1}^{m}R\left(s,m-k\right)\left[A\left(s,k\right)-B\left(s,k\right)\right],$$

(31) $$z\left(s,m,n\right)=-\sum_{k=1}^{m}R\left(s,m-k\right)y\left(s,k,n\right),$$

and inserting (25) and (29) into (26) yields:

(32) p(s,m,n)=X(s,m)p(s,1,n)+z(s,m,n),m=2,…,N.

Now, combining (23) with (26), we can compute p(s,1,n), whereas p(s,0,n) can be derived from (21). These results can be gathered in the final theorem.

**Theorem** **1.** *The transform of the queue size distribution at t in the GI/MSP/1/N system is:*

(33) $$\begin{array}{cc} p(s,1,n)= & {\left[X\left(s,N\right)-A^{*}\left(s\right)B\left(s,N\right)-A^{*}\left(s\right)\sum_{l=1}^{N-1}A\left(s,l\right)X\left(s,N-l+1\right)\right]}^{-1}\\ \; & \cdot \left[A^{*}\left(s\right)\sum_{l=1}^{N-1}A\left(s,l\right)z\left(s,N-l+1,n\right)+A^{*}\left(s\right)y\left(s,N,n\right)-z\left(s,N,n\right)\right], \end{array}$$

(34) p(s,0,n)=g(s)p(s,1,n)+w(s,n),

(35) p(s,m,n)=X(s,m)p(s,1,n)+z(s,m,n),m=2,…,N,

*where*

(36) $$A^{*}\left(s\right)={\left[I_{M}-A\left(s,0\right)\right]}^{-1},$$

*where $I_{M}$ is the identity matrix, whereas g(s), A(s,l), B(s,l), y(s,m,n), w(s,n), X(s,m), and z(s,m,n), are given in (7), (8), (9), (20), (22), (30), and (31), respectively.*

### 4.2. Mean Queue Length

In this section, we seek to determine the mean queue length at *t*, given that at the time origin the queue length was *m* and the service phase was *j*. Namely, we want to derive:

(37) $$Q_{j}\left(t,m\right)=E\left\{X\left(t\right)|X\left(0\right)=m,S\left(0\right)=j\right\},\;\;\;\;m=0,\ldots ,N;j=1,\ldots ,M.$$

Naturally, the mean queue length can be calculated from the queue length distribution, i.e., via Theorem 1. This approach, however, requires calculating N+1 queue length probabilities from Formulas (33)–(35). Apparently, this is not necessary if we are interested only in the mean value, instead of the complete distribution.

Below, we show how the mean value can be obtained faster, without computation of the complete distribution in the intermediate step. The idea is to design analogs of Equations (3)–(5) directly for $Q_{j}\left(t,m\right)$, rather than for $P_{j}\left(t,m,n\right)$.

Specifically, for m=1,…,N−1 and any *j*, we have:

(38) $$\begin{array}{cc} Q_{j}\left(t,m\right)= & \sum_{l=0}^{m-1}\sum_{k=1}^{M}\int_{0}^{t}f_{jk}\left(u,l\right)Q_{k}\left(t-u,m-l+1\right)dG\left(u\right)\\ \; & +\sum_{k=1}^{M}\int_{0}^{t}r_{jk}\left(u,m\right)Q_{k}\left(t-u,1\right)dG\left(u\right)\\ \; & +\left[1-G\left(t\right)\right]\sum_{n=0}^{m-1}\sum_{k=1}^{M}\left(m-n\right)f_{jk}\left(t,n\right). \end{array}$$

This equation is obtained in a similar fashion as (3), with obvious changes.

Then, for any *j*, we have:

(39) $$\begin{array}{c} Q_{j}\left(t,0\right)=\int_{0}^{t}Q_{j}\left(t-u,1\right)dG\left(u\right), \end{array}$$

which is built in a similar manner as (4) and

(40) $$\begin{array}{cc} Q_{j}\left(t,N\right)= & \sum_{k=1}^{M}\int_{0}^{t}f_{jk}\left(u,0\right)Q_{k}\left(t-u,N\right)dG\left(u\right)\\ \; & +\sum_{l=1}^{N-1}\sum_{k=1}^{M}\int_{0}^{t}f_{jk}\left(u,l\right)Q_{k}\left(t-u,N-l+1\right)dG\left(u\right)\\ \; & +\sum_{k=1}^{M}\int_{0}^{t}r_{jk}\left(u,N\right)Q_{k}\left(t-u,1\right)dG\left(u\right)\\ \; & +\left[1-G\left(t\right)\right]\sum_{n=0}^{N-1}\sum_{k=1}^{M}\left(N-n\right)f_{jk}\left(t,n\right), \end{array}$$

which is built in a similar manner as (5).

The system (38)–(40) has the same general form as the system (3)–(5). Therefore, it can be solved in an analogous manner as (3)–(5).

Defining:

(41) $$q_{j}\left(s,m\right)=\int_{0}^{\infty }e^{-st}Q_{j}\left(t,m\right)dt,$$

(42) $$q\left(s,m\right)={\left[q_{1}\left(s,m\right),\ldots ,q_{M}\left(s,m\right)\right]}^{T},$$

and solving (38)–(40), we acquire the following theorem.

**Theorem** **2.** *The transform of the mean queue length at t in the GI/MSP/1/N system is:*

(43) $$\begin{array}{cc} q(s,1)= & {\left[X\left(s,N\right)-A^{*}\left(s\right)B\left(s,N\right)-A^{*}\left(s\right)\sum_{l=1}^{N-1}A\left(s,l\right)X\left(s,N-l+1\right)\right]}^{-1}\\ \; & \cdot \left[A^{*}\left(s\right)\sum_{l=1}^{N-1}A\left(s,l\right)x\left(s,N-l+1\right)+A^{*}\left(s\right)v\left(s,N\right)-x\left(s,N\right)\right], \end{array}$$

(44) q(s,0)=g(s)q(s,1),

(45) q(s,m)=X(s,m)q(s,1)+x(s,m),m=2,…,N,

where g(s), A(s,l), B(s,l), X(s,m), and $A^{*}\left(s,l\right)$, are given in (7), (8), (9), (30), and (36), respectively, while

(46) $$v\left(s,m\right)=\sum_{n=0}^{m-1}\left(m-n\right)C\left(s,n\right)1,$$

(47) $$x\left(s,m\right)=-\sum_{k=1}^{m}R\left(s,m-k\right)v\left(s,k\right).$$

## 5. Auxiliary Results

In order to use Theorems 1 and 2 in numerical computations, we need matrices A(s,l), B(s,l), and C(s,l) defined in (8)–(10) and (18)–(16). All the remaining vectors and matrices in Theorems 1 and 2 are functions of those three.

Matrices A(s,l) and C(s,l) are easy to compute using the well-known uniformization technique (see, e.g., [18], p. 66).

What still needs to be performed is to calculate B(s,l). To accomplish that, define:

(48) $$F\left(t,l\right)={\left[f_{jk}\left(t,l\right)\right]}_{j=1,\ldots ,M;k=1,\ldots ,M},$$

(49) $$R^{*}\left(t,l\right)={\left[r_{jk}\left(t,l\right)\right]}_{j=1,\ldots ,M;k=1,\ldots ,M},$$

(50) $$H\left(s,l\right)=\int_{0}^{\infty }e^{-st}R^{*}\left(t,l\right)\left[1-G\left(t\right)\right]dt,$$

(51) $$\bar{g}\left(s\right)=\int_{0}^{\infty }e^{-st}\left(1-G\left(t\right)\right)dt.$$

It is known that:

(52) $$\frac{d}{dt}F\left(t,l\right)=F\left(t,l\right)L_{0}+F\left(t,l-1\right)L_{1},\;\;\;l\geq 1,$$

(53) $$F\left(t,0\right)=e^{L_{0}t},$$

(54) $$\frac{d}{dt}R^{*}\left(t,l\right)=F\left(t,l-1\right)L_{1},\;\;\;l\geq 1,$$

see Formulas (2), (4) and (11) in [10], respectively.

Therefore, using (54), we have:

(55) $$\begin{array}{cc} B(s,l) & =\int_{0}^{\infty }e^{-st}R^{*}\left(t,l\right)dG\left(t\right)=-\int_{0}^{\infty }e^{-st}R^{*}\left(t,l\right)\left[1-G\left(t\right)\right]\\ \; & =-e^{-st}R^{*}\left(t,l\right)\left[1-G\left(t\right)\right]{|}_{0}^{\infty }+\int_{0}^{\infty }\frac{d}{dt}\left[e^{-st}R^{*}\left(t,l\right)\right]\left[1-G\left(t\right)\right]dt\\ \; & =\int_{0}^{\infty }e^{-st}F\left(t,l-1\right)L_{1}\left[1-G\left(t\right)\right]dt-s\int_{0}^{\infty }e^{-st}R^{*}\left(t,l\right)\left[1-G\left(t\right)\right]dt\\ \; & =C\left(s,l-1\right)L_{1}-sH\left(s,l\right),\;\;\;\;\;\;l\geq 1. \end{array}$$

As we can see, B(s,l) is a function of H(s,l), which is also unknown. To compute H(s,1), we can exploit (53) and (54). We have:

(56) $$\begin{array}{cc} H(s,1) & =\int_{0}^{\infty }e^{-st}R^{*}\left(t,1\right)\left[1-G\left(t\right)\right]dt=\int_{0}^{\infty }e^{-st}\left[1-G\left(t\right)\right]dt\int_{0}^{t}F\left(u,0\right)L_{1}du\\ \; & =\int_{0}^{\infty }e^{-st}\left[1-G\left(t\right)\right]dt\int_{0}^{t}e^{L_{0}u}L_{1}du=\int_{0}^{\infty }e^{-st}\left(e^{L_{0}t}-I_{M}\right)\left[1-G\left(t\right)\right]dtL_{0}^{-1}L_{1}\\ \; & =\int_{0}^{\infty }e^{-st}F\left(t,0\right)\left[1-G\left(t\right)\right]dtL_{0}^{-1}L_{1}-\int_{0}^{\infty }e^{-st}\left[1-G\left(t\right)\right]dtL_{0}^{-1}L_{1}\\ \; & =C\left(s,0\right)L_{0}^{-1}L_{1}-\bar{g}\left(s\right)L_{0}^{-1}L_{1}. \end{array}$$

Similarly, using (52) and (54), we get:

(57) $$\begin{array}{cc} H(s,l+1) & =\int_{0}^{\infty }e^{-st}\left[1-G\left(t\right)\right]dt\int_{0}^{t}F\left(u,l\right)L_{1}du\\ \; & =\int_{0}^{\infty }e^{-st}\left[1-G\left(t\right)\right]dt\int_{0}^{t}\left[\frac{d}{du}F\left(u,l\right)-\frac{d}{du}R^{*}\left(u,l\right)\right]duL_{0}^{-1}L_{1}\\ \; & =C\left(s,l\right)L_{0}^{-1}L_{1}-H\left(s,l\right)L_{0}^{-1}L_{1},\;\;\;\;\;l\geq 1. \end{array}$$

Now, Formula (55), accompanied with (56) and (57), can be exploited to calculate B(s,l) for arbitrary *l*.

The presented calculations of B(s,l) are based on a similar technique to the technique used in the computation of $\Omega_{n}$ in [12]. They are slightly more complicated here, due to the additional variable *s*, which is absent in [12].

Having all the components of Theorems 1 and 2, we can compute p(s,m,n) and q(s,m) and invert them to the time domain, by means of an inversion method for the Laplace transform. In the numerical examples here, we use the method of [19].

## 6. Numerical Results

In these numerical examples, we will use three parameterizations of the MSP, which differ in the service correlation strength but share a common service rate. Namely, we will use:

- $MSP_{1}$:
  (58) $$L_{0}=\left[\begin{array}{ccc} -17.546000 & 17.161520 & 0.347432\\ 10.681103 & -11.534700 & 0.341986\\ 0.428089 & 0.044159 & -2.418643 \end{array}\right],$$
  (59) $$L_{1}=\left[\begin{array}{ccc} 0.008499 & 0.015647 & 0.012902\\ 0.502039 & 0.003184 & 0.006388\\ 0.018356 & 0.002029 & 1.926010 \end{array}\right],$$
- $MSP_{2}$:
  (60) $$L_{0}=\left[\begin{array}{ccc} -3.993320 & 3.887300 & 0.068972\\ 2.038960 & -2.623340 & 0.072769\\ 0.083084 & 0.008466 & -2.037945 \end{array}\right],$$
  (61) $$L_{1}\mathrm{is}\;\mathrm{the}\;\mathrm{same}\;\mathrm{as}\;\mathrm{in}\;MSP_{1},$$
- $MSP_{3}$:
  (62) $$L_{0}=\left[\begin{array}{ccc} -0.963063 & 0.919304 & 0.006711\\ 0.106657 & -0.630842 & 0.012574\\ 0.005944 & 0.000485 & -1.952824 \end{array}\right],$$
  (63) $$L_{1}\mathrm{is}\;\mathrm{the}\;\mathrm{same}\;\mathrm{as}\;\mathrm{in}\;MSP_{1}.$$

**Figure 1:**
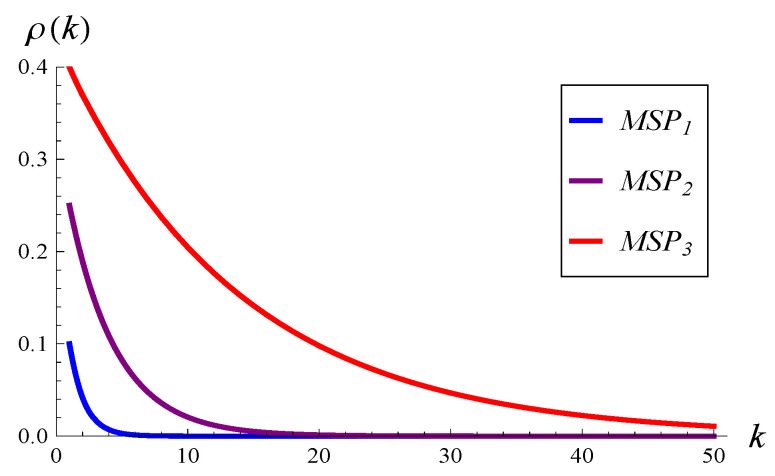
The autocorrelation of service times in $MSP_{1}$, $MSP_{2}$, and $MSP_{3}$ versus lag.

All three have the same service rate of 1. However, the 1-lag correlation in $MSP_{1}$, $MSP_{2}$, and $MSP_{3}$ is 0.1, 0.25, and 0.4, respectively. Moreover, the correlation in $MSP_{2}$ decays more slowly with lag than that in $MSP_{1}$. The correlation in $MSP_{3}$ decays even more slowly than that of $MSP_{2}$. Therefore, we will conventionally refer to $MSP_{1}$ as ’weakly correlated’, $MSP_{2}$ as ’moderately correlated’, and $MSP_{3}$ as ’strongly correlated’. In Figure 1, the autocorrelation functions for all three MSPs are depicted. They were obtained using Formula (2.14) of [18], p. 71.

If not stated otherwise, the interarrival time distribution will be as follows:

(64) $$G\left(t\right)=1-0.2154e^{-0.4t}-0.7846e^{-1.7t},\;\;\;\;\;t>0.$$

It has a mean of 1 and a variance of 2.24, i.e., significantly larger than that in the Poisson process. Accordingly, the arrival process is strongly non-Poisson (distribution (64) will be altered at the close of this section only).

The waiting room capacity is N=20 and remains unchanged in all the examples.

**Figure 2:**
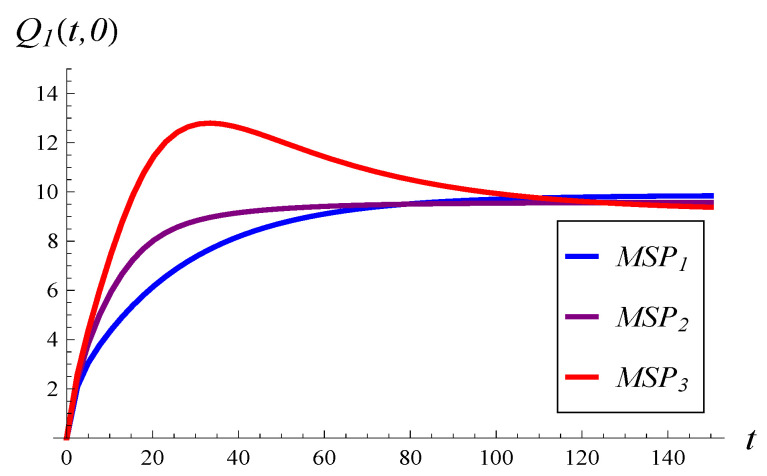
Mean queue length versus time for the three MSPs and X(0)=0, S(0)=1.

**Figure 3:**
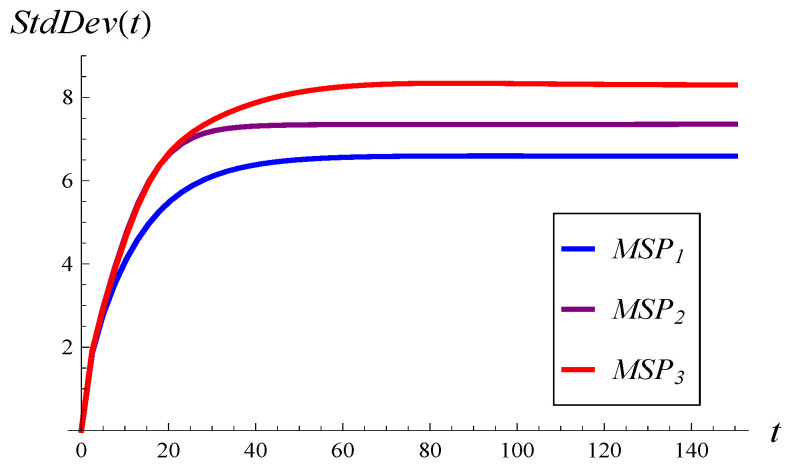
Queue length standard deviation versus time for the three MSPs and X(0)=0, S(0)=1.

In Figure 2, the development of the mean queue length over time is shown for the three MSPs, assuming an initially empty queue and an initial service phase of 1. Similarly, in Figure 3, the development of the standard deviation over time is depicted.

As we can see, a stronger correlation in service times induces a larger standard deviation for every *t* (see Figure 3) and a more irregular (non-monotonic) development of the mean value (see Figure 2).

It is worth noting that, with strong correlation in service times, the standard deviation is quite close to the maximum possible value. For instance, in Figure 3, the standard deviation for $MSP_{3}$ grows to 8.29 over time, while the maximum possible value for any distribution is 10.

The mean queue length converges to the following stationary values in Figure 3: 9.84, 9.56, and 9.16 for the weakly, moderately, and strongly correlated MSP, respectively. This is rather unexpected, as one might expect the stationary mean length to grow with the service correlation strength, rather than to decrease.

**Figure 4:**
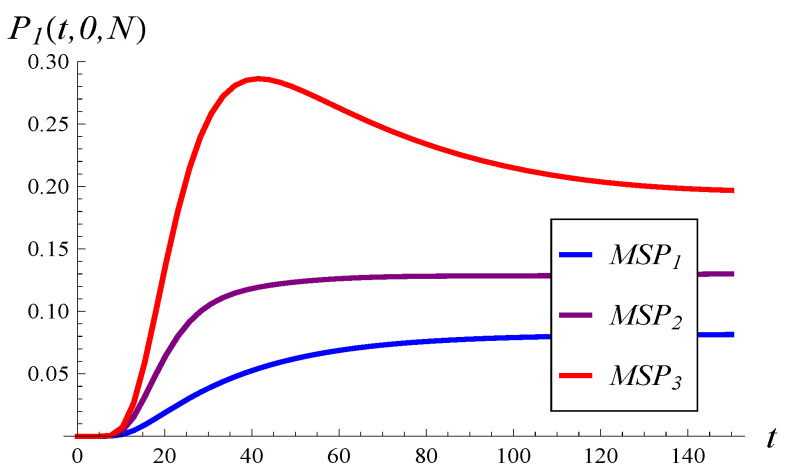
Blocking probability versus time for the three MSPs and X(0)=0, S(0)=1.

This phenomenon can be understood by studying Figure 4. Specifically, in Figure 4, the probability that the waiting room is saturated at time *t* is depicted for the three MSPs of interest. As we can see, the stronger the correlation, the higher the blocking probability, and this holds true for every t∈(0,∞). Therefore, as the correlation becomes stronger, more customers are prohibited from entering the queue, which reduces the effective load on the queue. Consequently, the stationary mean queue length decreases.

**Figure 5:**
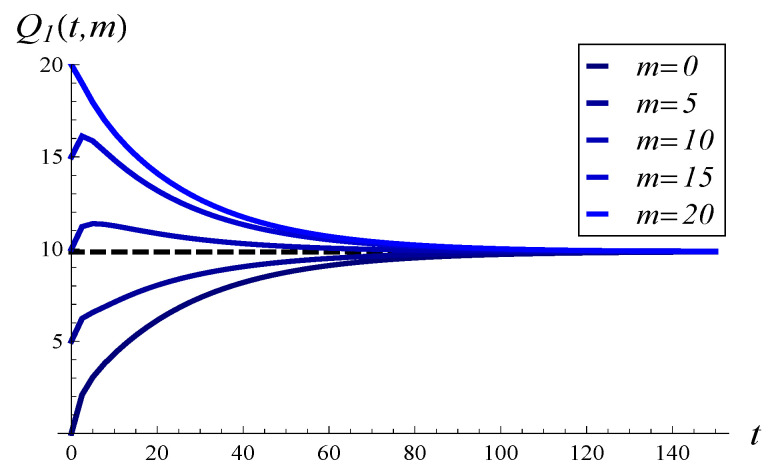
Mean queue length versus time for $MSP_{1}$ and several values of m=X(0).

**Figure 6:**
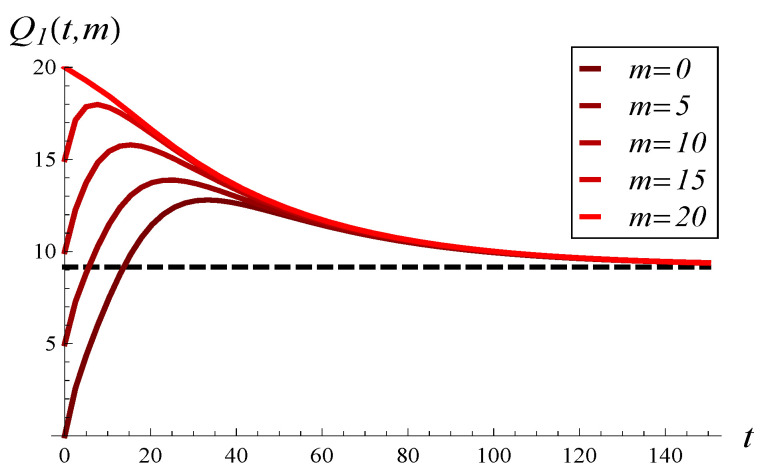
Mean queue length versus time for $MSP_{3}$ and several values of m=X(0).

In Figure 5, the development of the mean queue length over time is depicted for $MSP_{1}$ and several initial queue lengths. In Figure 6, $MSP_{1}$ is replaced with $MSP_{3}$. In both figures, the dashed line represents the stationary value of the mean queue length.

Surprisingly, the initial queue length seems to have a small effect on the time to attain the stationary regime in both figures. In Figure 5, the convergence time is perhaps slightly shorter for X(0)=10 than for X(0)=0 or X(0)=N, but the difference is not significant. In the case of strongly correlated service, shown in Figure 6, the initial queue length has no impact at all on the convergence time. Specifically, the time to attain stationary regime is the same for all initial lengths. The correlation itself has a much deeper impact, so all the lines in Figure 6 overlap starting from around t=60, well before the stationary regime is attained.

When comparing Figure 5 with Figure 6, it is clear that stronger service correlation in Figure 6 induces a longer convergence time to the stationary value.

This will be even more apparent in the full transient queue length distributions, shown below.

**Figure 7:**
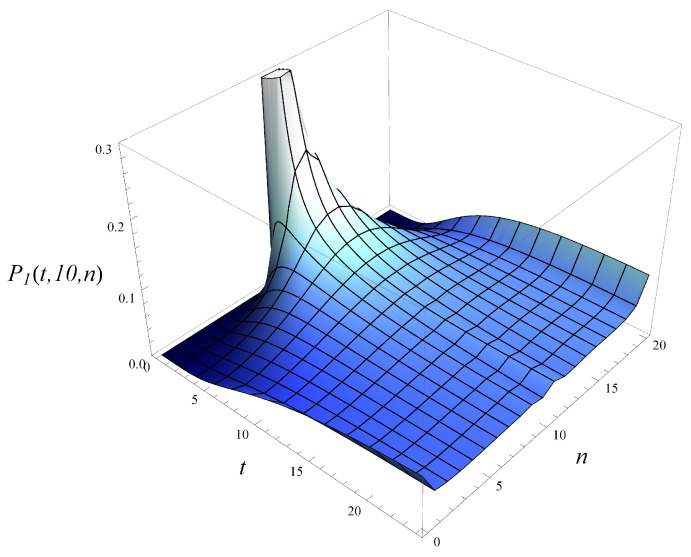
Queue length distribution versus time for $MSP_{1}$ and X(0)=10, S(0)=1.

**Figure 8:**
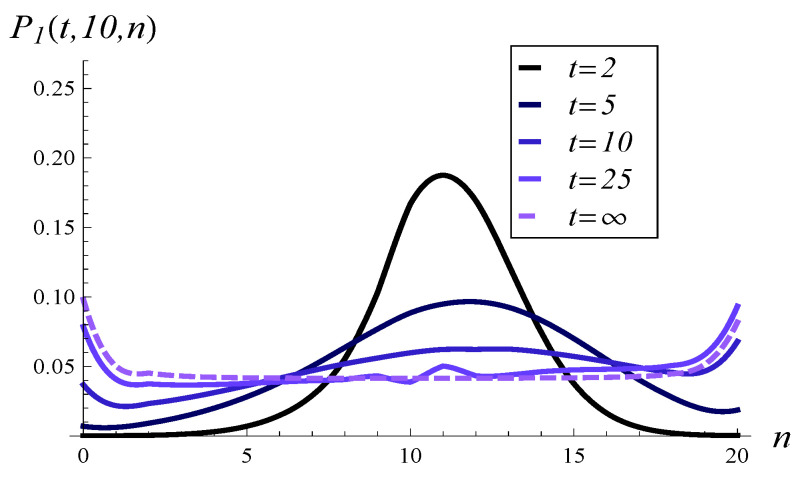
Queue length distribution at selected moments in time for $MSP_{1}$ and X(0)=10, S(0)=1.

Now, in Figure 7, the development of the queue length distribution over time is depicted for $MSP_{1}$ with an initial queue length of 10. Figure 8 shows a few vertical slices of Figure 7, representing the queue length distribution at selected moments: t=2, t=5, t=10, t=25, and t=∞.

**Figure 9:**
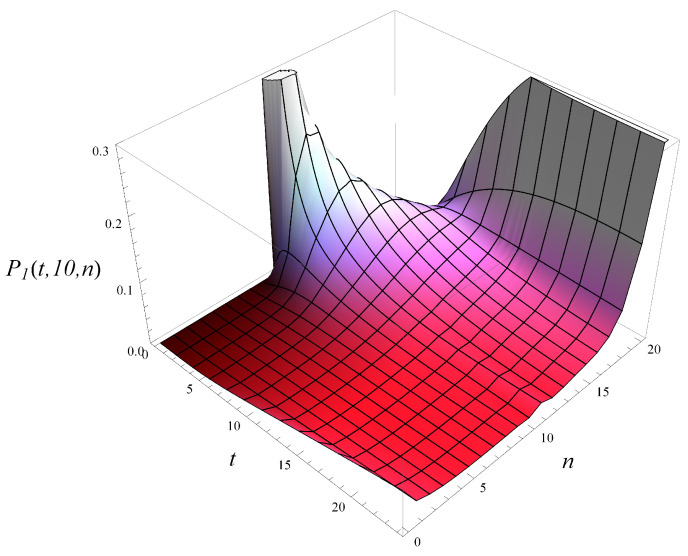
Queue length distribution versus time for $MSP_{3}$ and X(0)=10, S(0)=1.

**Figure 10:**
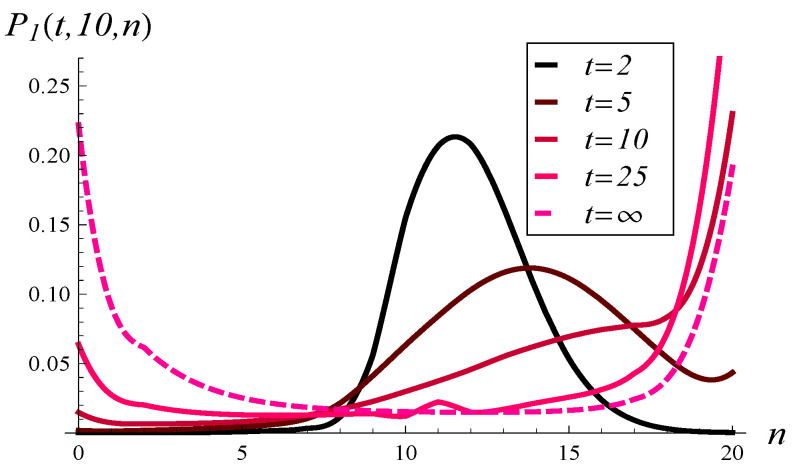
Queue length distribution at selected moments in time for $MSP_{3}$ and X(0)=10, S(0)=1.

Similarly, in Figure 9, the development of the queue length distribution over time is depicted for $MSP_{3}$ with an initial queue length of 10, while Figure 10 shows a few vertical slices of Figure 9.

When analyzing Figure 7, Figure 8, Figure 9 and Figure 10, we observe a much slower convergence to the stationary distribution in the case of $MSP_{3}$ compared to $MSP_{1}$. Specifically, for $MSP_{1}$, the queue length distribution at t=25 is already quite close to the stationary distribution (see Figure 8). In contrast, Figure 10 shows that at t=25, the queue length distribution for $MSP_{3}$ is still far from the stationary distribution. This is attributable to the strong service correlation in $MSP_{3}$.

Moreover, in Figure 10, we see that a high probability mass is concentrated at lengths 0 and *N* in the stationary regime. This concentration explains the high standard deviation observed for $MSP_{3}$ in Figure 3.

Previously, we have altered only the service process and initial conditions, while keeping the interarrival distribution provided in (64), with a variance of 2.24. For comparison, we will also use Poisson arrivals with a rate of 1 and a variance of 1, and the hypoexponential interarrival time with parameters:

(65) $$\left(\lambda_{1},\lambda_{2},\lambda_{3}\right)=\left(3.1250,3.0303,2.9412\right),$$

which provide a rate of 1 and a variance of 0.33. All three distributions of the interarrival time, i.e., (64), Poisson, and hypoexponential, will be used with the strongly correlated service, $MSP_{3}$.

**Figure 11:**
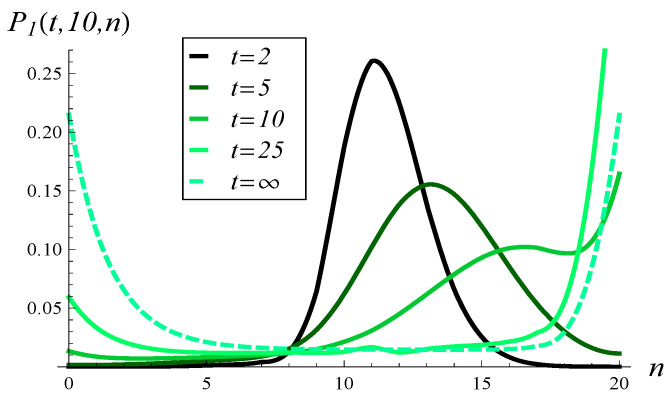
Queue length distribution at selected moments in time for $MSP_{3}$, Poisson arrivals, and X(0)=10, S(0)=1.

**Figure 12:**
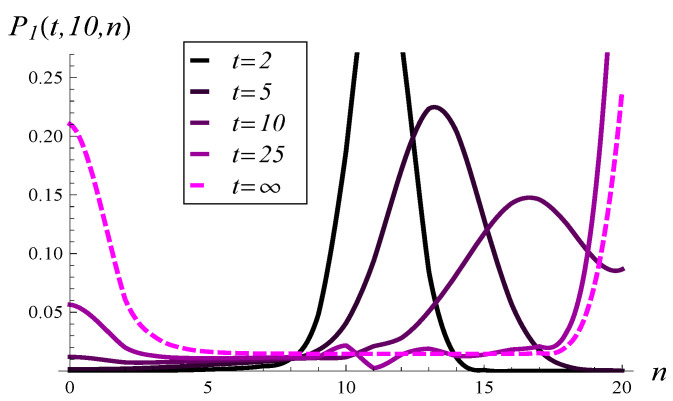
Queue length distribution at selected moments in time for $MSP_{3}$, hypoexponential arrivals, and X(0)=10, S(0)=1.

In Figure 11 and Figure 12, queue length distributions at selected moments in time for Poisson and hypoexponential arrivals are depicted, respectively. These two figures should be compared with Figure 10, which shows results for the same service process but arrivals given by (64). As we can see in Figure 10, Figure 11 and Figure 12, for small values of *t*, the queue length distribution forms a higher peak the smaller the interarrival variance is.

**Figure 13:**
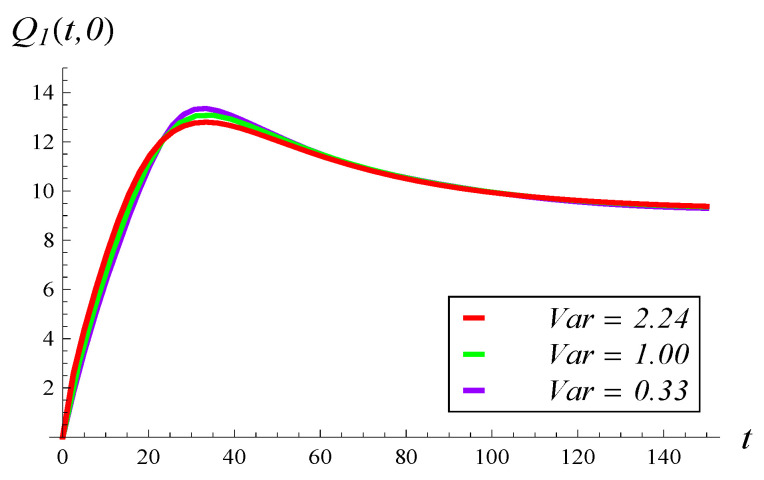
Mean queue length versus time for three interarrival variances. $MSP_{3}$, X(0)=0, S(0)=1.

In Figure 13, the development of the mean queue length over time is depicted for $MSP_{3}$ with the three arrival processes: (64), Poisson, and hypoexponential. As we can see, the curves practically overlap. Clearly, the strong correlation in $MSP_{3}$ has a greater impact on the mean queue length than the interarrival variance, at least when this variance ranges from 0.33 to 2.24. To obtain more distinct curves in Figure 13, a much larger difference in the interarrival variance would be required, such that it would exceed the impact of the strong service correlation.

## 7. Conclusions

We carried out the transient study of the queue length distribution in a model with the Markovian service process, a general distribution of interarrival times, and a finite waiting room. The Markovian service process was chosen because it is one of the most attractive and analytically tractable models for correlated service times, a phenomenon that is present in many real queues and has a major effect on their performance.

Two theorems were proven in the paper: one on the distribution of the queue length at a particular time *t*, where *t* can be arbitrarily small or large, and another on the mean queue length at *t*. In addition to the theorems, several numerical examples were provided and discussed. These examples allowed us to observe the development over time of the mean queue length, along with its standard deviation and complete distribution, depending on the service correlation strength, initial system conditions, and the interarrival variance.

For instance, we observed that a strong correlation in service times resulted in a longer convergence to the stationary distribution. We also saw that strong correlation increased the standard deviation of the queue, making it close to the maximum possible value. Additionally, we encountered and explained the unexpected phenomenon that the stationary mean queue length was shorter with strong service correlation compared to cases with weak or moderate correlation.

Future work could focus on the transient analysis of models with an infinite waiting room, or models expanded to include batch arrivals, batch services, or both.

## Data Availability

Data is contained within the article.
